# The Knowledge and Attitude of Parents About the Dental Treatment of Their Children During the New Type of Coronavirus Outbreak in Northern Cyprus

**DOI:** 10.3389/fpubh.2022.821474

**Published:** 2022-02-14

**Authors:** Ayse Ekinci, Ozgur Tosun, Aylin Islam

**Affiliations:** ^1^Department of Pediatric Dentistry, Near East University, Nicosia, Cyprus; ^2^Department of Biostatistics, Near East University, Nicosia, Cyprus

**Keywords:** COVID-19, pediatric dentistry, attitude, knowledge, dental treatment

## Abstract

**Background:**

The aim of this study was to assess the knowledge and attitude of parents living in Northern Cyprus about the oral care and dental treatments of their children during the outbreak of the new type of Coronavirus (COVID-19).

**Methods:**

An online self-administered questionnaire was conducted consisting of 33 questions. A total of 256 parents participated in this study. The questionnaire was divided into two parts. The first part consists of demographic information and the second part consists of the awareness and knowledge of parents about dental treatments during COVID-19. SPSS software was used for statistical data analysis.

**Results:**

In total, 81.9% of the mothers and 59.6% of the fathers stated that their children could be infected with COVID-19 during dental treatments and were apprehensive about their children undergoing such treatment because of the outbreak. Participants were asked about their knowledge regarding the transmission of COVID-19 through air droplets during dental treatments and the findings revealed that mothers (89.5%) were more aware of this than fathers (77.2%). The majority of the participants (77%) stated that their children could become infected with COVID-19 during dental treatments and were apprehensive about their children undergoing such treatment because of the outbreak; however, 65.1% did not take any extra precautions regarding their children's oral health and care.

**Conclusion:**

The majority of the parents in Northern Cyprus have good knowledge about dental clinics being one of the high-risk areas where COVID-19 can be transmitted by aerosols from infected people. However, further steps need to be taken to strengthen parents' motivations for home oral care and more studies are needed to assess the continuing impact of the COVID-19 pandemic on parents' attitudes toward and knowledge about dental procedures.

## Introduction

The first case of severe acute respiratory syndrome Coronavirus 2 (SARS-Cov-2) or Coronavirus disease 2019 (COVID-19) was reported in a patient in Wuhan, China, in late 2019 (1). Following the first case and the rapid spread of the virus, the World Health Organization (WHO) soon announced that the COVID-19 outbreak had become an international public health crisis and categorized it as a pandemic in March 2020. The number of cases and deaths caused by the virus is continually increasing around the world and the total number of confirmed cases reported by national authorities has surpassed 202 million, while the number of deaths caused by COVID-19 is now over 4.3 million (2).

Coronaviruses (CoVs) comprise a group of related viruses that includes Human Coronavirus 229E (HCoV-229E), Human Coronavirus OC43 (HCoV-OC43), Human Coronavirus NL63 (HCoV- NL63), and Human Coronavirus HKU1 (HCoV-HKU1), which can cause respiratory tract infections in humans ranging from mild symptoms to fatal outcomes. The remaining three CoVs genera, which are Severe Acute Respiratory Syndrome Coronavirus (SARS-CoV), Middle East Respiratory Syndrome Coronavirus (MERS- CoV), and Severe Acute Respiratory Syndrome Coronavirus 2 (SARS-CoV-2), are highly pathogenic and may lead to severe respiratory diseases with a possibly fatal outcome in infected patients (1). In September 2020, a new variant with a higher propagation rate named Alpha (B.1.1.7) was detected in the United Kingdom on September, followed by a second variant called Beta (B.1.351/501.YV2) in South Africa on May 2020, and the third variant was identified in a Brazilian tourist in Japan on November 2020, which was named Gamma (B.1.1.248/B1.1.28/P1). The fourth variant was the Delta (B.1.617.2) variant seen in India in November 2020, and finally, in November 2021, the fifth variant called Omicron (B.1.1.529) was announced by the World Health Organization (3). Omicron is the variant with the highest number of mutations among all COVID-19 variants identified thus far. There are 32 mutations in the spike protein, which determines the infectivity and antigenicity of the virus (4). All these variants have spread to other countries over time, and new variants are expected to emerge in the future (3).

Vaccination is a safe, simple and effective way of protecting people and in December 2020, just 11 months after the disease first emerged, more than 150 official vaccine projects of the COVID-19 genome were developed (5). In May 2021, the WHO reported that 1.26 billion doses of SARS-CoV-2 vaccines had been administered globally, and at least 637 million people had their first dose of vaccines. One lakh cumulative cases were recorded in nine African countries. Ensuring that all global citizens are offered the vaccine they need will provide a remarkable return on investment and help keep the world safe from future pandemics (6).

There are four main transmission pathways of COVID-19: symptomatic transmission (from a COVID-19 positive patient), pre-symptomatic transmission (from infected people who have not yet developed symptoms but go on to develop symptoms later), asymptomatic transmission (direct transmission from infected people but never develop any symptoms), and environmental transmission (indirect transmission that is not detectable) (7).

Also, the spread of infection may occur directly *via* air droplet transmission or through direct and indirect physical contact. Exposure to respiratory droplets produced when coughing, breathing, talking, or to the blood of a person with COVID-19 may lead to infection when inhaled or deposited on mucous membranes such as the nose, mouth, or eyes. Additionally, the main entrance of the virus occurs *via* the Angiotensin-converting enzyme 2 (ACE2) cell receptors, which are present throughout the respiratory tract, gut, and heart (8).

Also, ACE2 has been found at high levels in the mucosa of the oral cavity, especially in the epithelial cells of the tongue (9). Dental interventions are also another important transmission pathway that poses a greater risk of infection for both dentists and patients. Water-cooled, high-speed hand tools could generate aerosols while dental procedures are being performed, and when these aerosols are combined with the body fluids in the oral cavity, such as blood and saliva, bio aerosols are generated (10). These bio aerosols are frequently contaminated with fungi, bacteria, viruses and float in the air for a long time, potentially being inhaled by dentists or patients (11).

The epidemiology and clinical manifestations of COVID-19 in children are still unclear. Although there have been case reports related to children and teens suggesting that they can be infected and become sick from COVID-19, the majority of these case reports have indicated that the symptoms of children are milder when compared to adults. COVID-19 occurs in three main stages in children: a mild cold-like illness, a moderate respiratory syndrome and a severe acute interstitial pneumonia (12). Several opinions have been put forward regarding the milder symptoms of the virus in children (13–15) one of these being that children tend to be exposed to various viruses in the community that often result in the common cold; therefore, this repeated viral exposure primes the immune system for greater protection against COVID-19. On the other hand, the reduced effects of COVID-19 in children are related to the immaturity of the ACE2 receptor and the decreased binding capacity of SARS CoV2 (13).

Regarding survey studies performed in the literature, we found similar studies regarding the knowledge of and attitudes toward COVID-19 among parents of child dental patients during the outbreak (16), Although studies including the Online Cross-Sectional Survey on Oral Healthcare Among School-Age Children During COVID-19 Epidemic in Wuhan, China (17), and Parents' Knowledge and Attitude toward COVID-19 in Children: A Jordanian Study (18) have been performed, it has been found that there are no previous studies on the knowledge and attitude of parents regarding their children‘s dental treatment during the COVID-19 pandemic in Northern Cyprus.

When the high risk of COVID-19 transmission during dental interventions is combined with milder symptoms / asymptomatic conditions in children, the significance of the pediatric dentistry field and the importance of oral hygiene in children during pandemics have come into prominence. Hence, the aim of this study was to investigate whether parents were sufficiently interested in their children's oral health during the COVID-19 pandemic when they were largely confined to their homes, and whether parents were aware of the risks of dental treatments in terms of contamination. Also, this study aimed to investigate whether parents knew that they should only visit the dentist for emergency treatments during the pandemic, as well as their level of knowledge about what conditions require emergency dentistry treatment. Additionally, popularization of tele-dentistry was aimed for the first time in Cyprus by using modern technology to raise public awareness regarding oral and dental health, as well as to facilitate guidance, education, or research without direct face-to-face contact with the patient.

## Materials and Methods

The current cross-sectional questionnaire was planned to be administered online to provide rapid access to parents during the pandemic. All parents participated voluntarily by completing a self-administered questionnaire in April and June 2020. During this period, Northern Cyprus was under complete lockdown. Prior to the online administration of the survey, the study was approved by the Near East University, Faculty of Medicine Ethical Review Board (NEU / 2020 / 78−1063). At the beginning of the online survey, the purpose of the study was explained and information was given indicating that individual results would not be disclosed to any other person or organization in order to ensure privacy. Subsequently, participants were required to complete a questionnaire that was designed to obtain information about the knowledge and attitudes of parents of pediatric patients regarding their dental treatments during the COVID-19 epidemic. Due to the fact that COVID-19 is a new disease and few studies have measured the level of knowledge of parents about the pandemic, the questionnaire was based on several questionnaires about COVID-19 and other contagious diseases used in previous similar studies (16, 19, 20).

### Sample Size Determination and Questionnaire Design

G^*^Power software was used for the calculation of the sample size. With a confidence level of 95% and a confidence interval of 7.5%, a targeted statistical power of 80% was calculated to be achieved with a sample size of 205. Considering the possible factors that could negatively affect the statistical power in survey studies, the decision was made that the size of the sample should be at least 20% higher than the calculated value. Additionally, considering the general population of Northern Cyprus, a total number of 256 surveys were completed.

This questionnaire was prepared to analyse information, attitudes and home measures to promote effective oral health management and disease prevention among children during the global pandemic. Inclusion criteria were determined as parents who lived in Northern Cyprus and had children in the 0–18 age group. Children with disabilities and other systemic diseases were excluded from the study because their parents might have had excessive concerns about dental treatment during the COVID-19 outbreak. In addition, children who had previously had COVID-19 were also excluded from the survey in terms of the validity of the information to be given.

The questionnaire comprised 33 self-prepared questions and was divided into two parts. The first part was composed of demographic information (the first nine questions): The survey participants were evaluated according to the parenting relationship. In terms of age groups, the parents were classified into three groups: <30, 30–40, and >40 years. Based on their education level, the parents were categorized as primary-high school and graduate-postgraduate. According to place of residence, parents were classified as either living in the city or the countryside and based on occupation, parents were evaluated in terms of whether they were health professionals or non-health professionals. Parents were categorized according to their minimum income level as follows: <3,323, 3,323–10,000, and >10,000 (Turkish lira). During this survey, 1 US dollar was equivalent to 6.5 Turkish liras. Therefore, parents' minimum income levels were as follows in US dollars: <511, 511–1.538, and >1.538. The second part included 24 questions. Of these, three questions were open-ended, 13 questions were multiple choices, and eight questions had possible answers of “yes” or “no.” The participants were allowed to proceed to the next question without answering the current question.

### Statistical Analysis

Answers obtained from this study were used descriptively. All answers were demonstrated in the form of percentages and frequencies. Chi-square tests were used for comparisons. All statistical tests were performed with Statistical Package for Social Sciences (SPSS) for Windows version 15.0 (SPSS Inc., Chicago, IL). The significance level was accepted at 0.05 for all statistical analyses.

## Results

A total of 256 parents participated in the study. 77.7% of the participants were mothers, 53.9% of the participants were in the 30–40 age group, 68.8% of the parents were graduates or postgraduates, the majority of the parents were living in the countryside (67.2%), the majority of the participants were non-health professionals (86.7) and 65.2% of the parents had an income of 511–1.538 US dollars. Table 1 shows the detailed demographic information of the participating parents.

**Table 1:** Sociodemographic features of the parents.

**Sociodemographic factors**	**Frequency**	**Percentage (%)**
**Parenthood relationship**		
Mother	159	77.7
Father	57	22.3
**Age groups (years)**		
<30	52	20.3
30–40	138	53.9
>40	66	25.8
**Level of education**		
Primary—High School	80	31.3
Graduate—Postgraduate	176	68.8
**Place of residence**		
City	84	32.8
Countryside	172	67.2
**Occupation**		
Health professionals	34	13.3
Non-health professionals	222	86.7
**Income (US dollars)**		
<511	35	13.7
511–1.538	167	65.2
>1.538	54	21.1

Table 2 includes the responses of the parents regarding whether children are more resistant to COVID-19. No statistically significant result was found, but when the findings were examined in detail, it was found that 67.8% of mothers thought that children are more resistant to COVID-19, while 32.2% did not agree with this statement. With respect to the fathers, 68.4% of them said that children are more resistant, while 31.6% thought that they are less resistant. Moreover, in the other comparison groups, a majority thought that children are more resistant to COVID-19.

**Table 2:** Responses of parents regarding whether children are more resistant to COVID-19.

**Sociodemographic factors**	**Yes (%)**	**No (%)**	**Chi-square**	***P*-value**
**Parenthood relationship**				
Mother	136 (67.8)	64 (32.2)	0.007	0.934
Father	39 (68.4)	18 (31.6)		
**Age groups (years)**				
<30	32 (61.5)	20 (38.5)	2.934	0.231
30–40	92 (66.7)	46 (33.3)		
>40	50 (75.8)	16 (24.2)		
**Level of education**				
Primary—High School	57 (71.3)	23 (28.8)	0.575	0.448
Graduate—Postgraduate	117 (66.5)	59 (33.5)		
**Place of residence**				
City	54 (64.3)	30 (35.7)	0.779	0.377
Countryside	120 (69.8)	52 (30.2)		
**Occupation**				
Health professionals	27 (79.4)	7 (20.6)	2.358	0.125
Non-health professionals	147 (66.2)	75 (33.8)		
**Income (US dollars)**				
<511	23 (65.7)	12 (34.3)	0.232	0.891
511–1.538	113 (67.7)	54 (32.3)		
>1.538	38 (70.4)	16 (29.6)		

The results of the comparison of the relationship between inadequate oral care in children and COVID-19 are given in Table 3, and statistically significant results were obtained in the age groups and occupations. While 57.7% of parents under the age of 30 thought that inadequate oral health care in their children were directly related to COVID-19, 42.3% reported that there was no relationship between them. Almost half of the participants (47.0%) over the age of 40 thought that there was a correlation, while the other half (53.0%) stated that inadequate oral care was not associated with COVID-19. On the other hand, only 27.5% of parents between the ages of 30 and 40 agreed with this statement, while 72.5% disagreed.

**Table 3:** Comparison of the relationship between inadequate oral care and COVID-19 in children.

**Sociodemographic factors**	**Yes (%)**	**No (%)**	**Chi-square**	***P*-value**
**Parenthood relationship**				
Mother	71 (35.7)	128 (64.3)	3.377	0.066
Father	28 (49.1)	29 (50.9)		
**Age groups (years)**				
<30	30 (57.7)	22 (42.3)	17.064	**0.000**
30–40	38 (27.5)	100 (72.5)		
>40	31 (47.0)	35 (53.0)		
**Level of education**				
Primary—High School	32 (40.0)	48 (60.0)	0.087	0.769
Graduate—Postgraduate	67 (38.1)	109 (61.9)		
**Place of residence**				
City	29 (34.5)	55 (65.5)	0.907	0.341
Countryside	70 (40.7)	102 (59.3)		
**Occupation**				
Health professionals	21 (61.8)	13 (38.2)	8.816	**0.003**
Non-health professionals	78 (35.1)	144 (64.9)		
**Income (US dollars)**				
<511	14 (40.0)	21 (60.0)	1.880	0.391
511–1.538	60 (35.9)	107 (64.1)		
>1.538	25 (46.3)	29 (53.7)		

*Bold shows statistical significance (p < 0.05)*.

**Figure 1:**
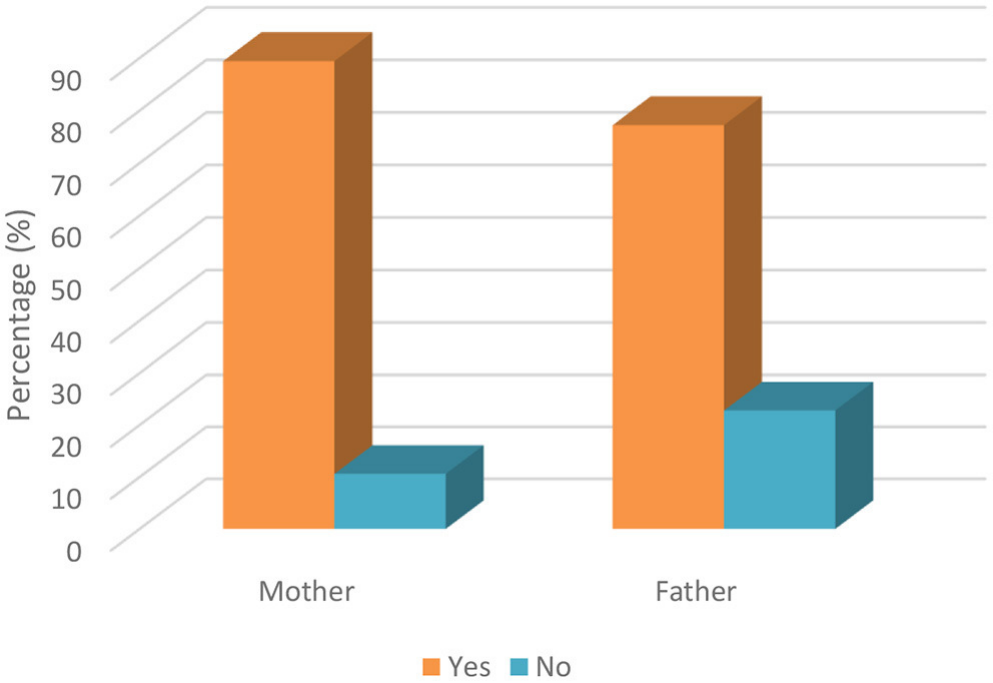
Knowledge of parents' about COVID-19 transmission by air droplets during dental treatment based on parenthood relationship.

**Figure 2:**
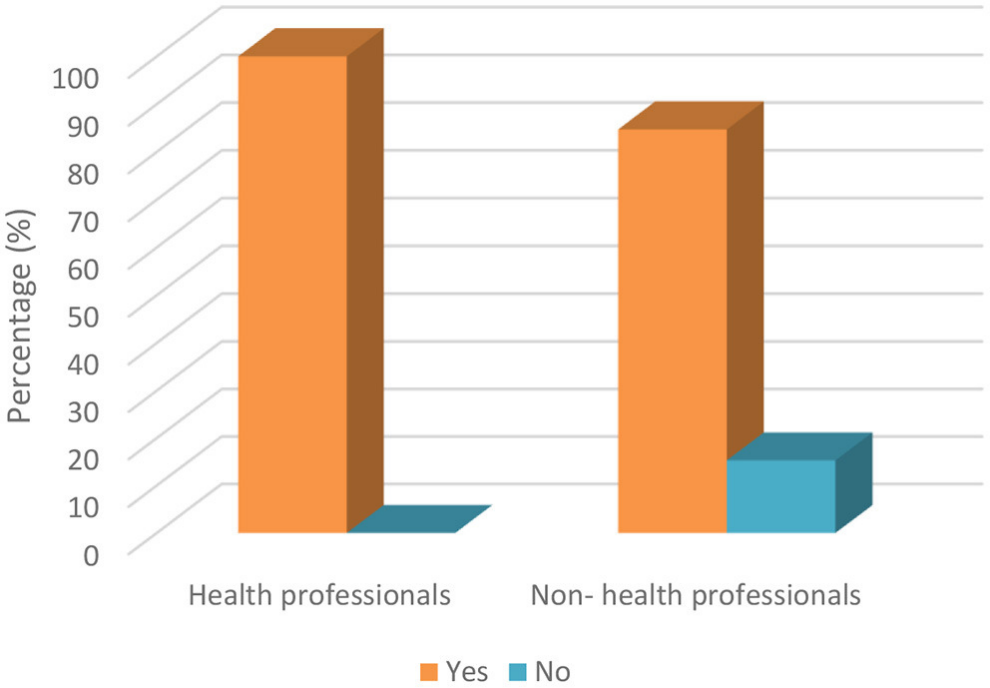
Knowledge of parents' about COVID-19 transmission by air droplets during dental treatment based on occupation.

The parents were asked whether they knew that dentistry clinics are one of the high-risk areas where COVID-19 can be transmitted through air droplets from infected individuals and that these droplets can remain in the air and on environmental surfaces for a long time. Table 4 shows the detailed relationship between sociodemographic factors and the transmission of COVID-19 by air droplets during dental treatments. The results reveal that there were significant differences in terms of the parenthood relationship and the occupation of the parents (*p* < 0.05). Mothers (89.5%) were more likely to be aware of this than fathers (77.2%) (Figure 1). Another important result was seen in occupational groups as we observed that 100% of health professionals stated that they knew that the virus can be transmitted by air droplets during dental treatments, while only 84.7% of other occupational groups had knowledge on this matter (Figure 2).

**Table 4:** Statistical analysis of parents' knowledge about COVID-19 transmission *via* air droplets during dental treatments based on sociodemographic factors.

**Sociodemographic factors**	**Yes (%)**	**No (%)**	**Chi-square**	***P*-value**
**Parenthood relationship**				
Mother	178 (89.4)	21 (10.6)	5.777	**0.016**
Father	44 (77.2)	13 (22.8)		
**Age groups (years)**				
<30	46 (88.5)	6 (11.5)	0.390	0.823
30–40	118 (85.5)	20 (14.5)		
>40	58 (87.9)	8 (12.1)		
**Level of education**				
Primary—High School	70 (87.5)	10 (12.5)	0.062	0.804
Graduate—Postgraduate	152 (86.4)	24 (13.6)		
**Place of residence**				
City	73 (86.9)	11 (13.1)	0.004	0.951
Countryside	149 (86.6)	23 (13.4)		
**Occupation**				
Health professionals	34 (100.0)	0 (0.0)		**0.012**
Non-health professionals	188 (84.7)	34 (15.3)		
**Income (US dollars)**				
<511	32 (91.4)	3 (8.6)	0.851	0.653
511–1.538	143 (85.6)	24 (14.4)		
>1.538	47 (87.0)	7 (13.0)		

*Bold shows statistical significance (p < 0.05)*.

**Figure 3:**
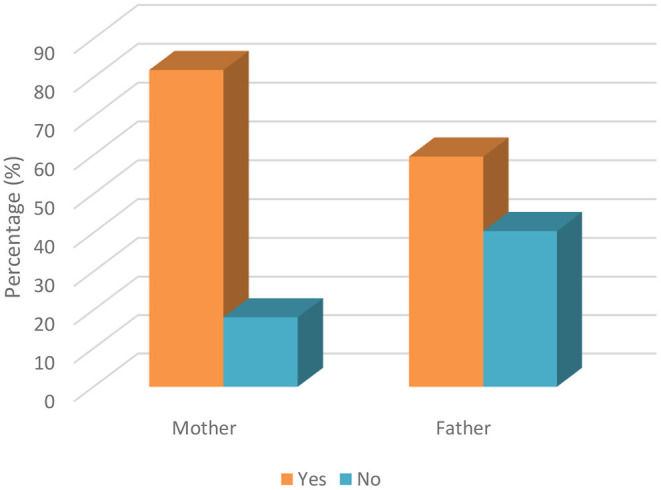
Parents' responses to whether they are concerned about taking their children to dental treatment during COVID-19 based on parenthood relationship.

Table 5 indicates the responses of parents regarding whether they were concerned about their children undergoing dental treatment during the COVID-19 pandemic. A statistically significant difference was observed between the responses of mothers and fathers; mothers were more worried about taking their children to the dentist during that period. While 81.9% of mothers were worried about taking their children to the dentist during the pandemic, 18.1% were not. On the other hand, while more than half of the fathers were worried (59.6%), the remainder (40.4%) were not. This statistical difference between parents was also represented in Figure 3. Also, another significant difference was found between ages. To elaborate on this further, while 90.4% of parents under the age of 30 were worried about taking their children to the dentist during this period, 9.6% stated that they were not worried. While 73.9% of parents between the ages of 30 and 40 were worried, 26.1% stated that they were not. However, while the 72.7% of parents over 40 reported being anxious, 27.3% reported that they were comfortable in this regard.

**Table 5:** Parents' responses regarding whether they were concerned about taking their children for dental treatment during COVID-19.

**Sociodemographic factors**	**Yes (%)**	**No (%)**	**Chi-square**	***P*-value**
**Parenthood relationship**				
Mother	163 (81.9)	36 (18.1)	12,380.0	**0.001**
Father	34 (59.6)	23 (40.4)		
**Age groups (years)**				
<30	47 (90.4)	5 (9.6)	6.673	**0.036**
30–40	102 (73.9)	36 (26.1)		
>40	48 (72.7)	18 (27.3)		
**Level of education**				
Primary—High School	67 (83.8)	13 (16.3)	3.031	0.082
Graduate—Postgraduate	130 (73.9)	46 (26.1)		
**Place of residence**				
City	62 (73.8)	22 (26.2)	0.697	0.404
Countryside	135 (78.5)	37 (21.5)		
**Occupation**				
Health professionals	29 (85.3)	5 (14.7)	1.538	0.215
Non- health professionals	168 (75.7)	54 (24.3)		
**Income (US dollars)**				
<511	30 (85.7)	5 (14.3)	1.760	0.415
511–1.538	126 (75.4)	41 (24.6)		
>1.538	41 (75.9)	13 (24.1)		

*Bold shows statistical significance (p < 0.05)*.

**Figure 4:**
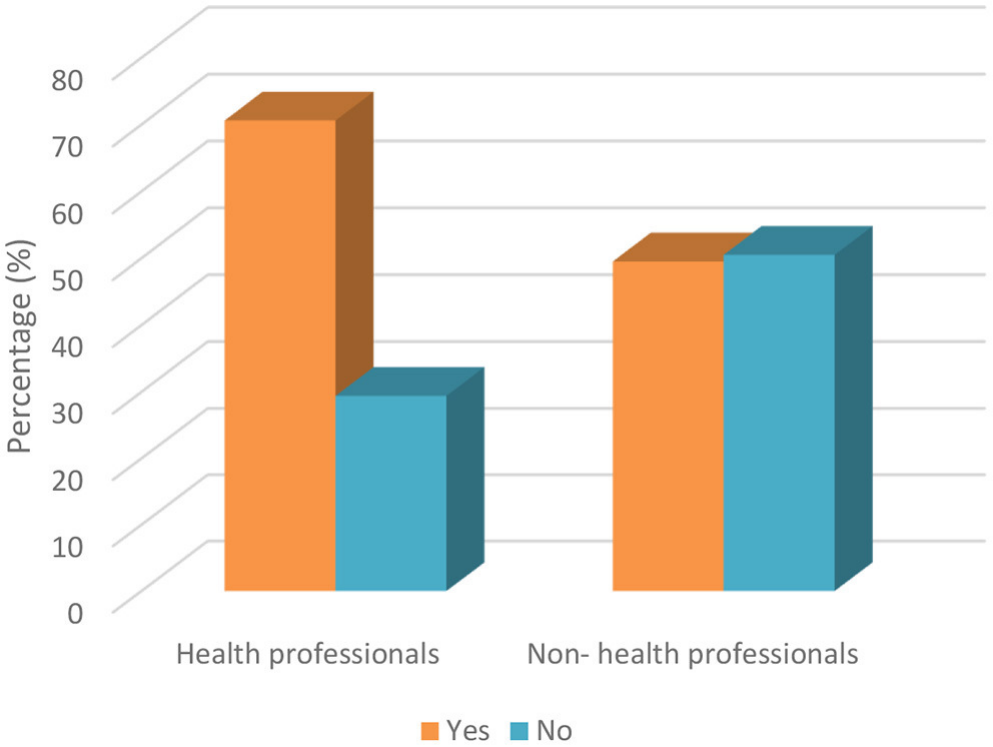
Awareness of participants about conditions requiring urgent dental treatment based on occupation.

The parents were asked about the conditions requiring urgent dental treatment. Significant differences were found between occupation groups and income ranges, as 70.6% of health professionals were aware of conditions requiring urgent dental treatment, while 49.5% of non-health professionals were aware of these conditions (Figure 4). It is certainly not a coincidence that healthcare professionals have a better level of knowledge on urgent treatments. Moreover, the percentage of parents who were knowledgeable about dental treatments according to income ranges of <511, 511–1.538, and >1.538 were 31.4, 19.8, and 9.3%, respectively. Parents with an income >1,538 US dollars had less knowledge about the conditions that require urgent dental treatment (Table 6).

**Table 6:** Awareness of parents about the conditions requiring urgent dental treatment.

**Sociodemographic factors**	**Yes (%)**	**No (%)**	**Chi-square**	***P*-value**
**Parenthood relationship**				
Mother	43 (21.6)	156 (78.4)	3.516	0.061
Father	6 (10.5)	51 (89.5)		
**Age groups (years)**				
<30	10 (19.2)	42 (80.8)	3.086	0.214
30–40	31 (22.5)	107 (77.5)		
>40	8 (12.1)	58 (87.9)		
**Level of education**				
Primary—High School	16 (20.0)	64 (80.0)	0.056	0.814
Graduate—Postgraduate	33 (18.7)	143 (81.3)		
**Place of residence**				
City	34 (19.8)	138 (80.2)	0.133	0.715
Countryside	15 (17.9)	69 (82.1)		
**Occupation**				
Health professionals	24 (70,6)	24 (70.6)	5.232	**0.022**
Non-health professionals	110 (49,5)	112 (50.5)		
**Income (US dollars)**				
<511	11 (31.4)	24 (68.6)	6.863	**0.032**
511–1.538	33 (19.8)	134 (80.2)		
>1.538	5 (9.3)	49 (90.7)		

*Bold shows statistical significance (p < 0.05)*.

Table 7 shows the responses given by parents on whether they would take any preventive measures for themselves and their children if they had to take their children to the dentist during the pandemic. The majority of mothers (80.9%) stated that they would take protective measures for themselves and their children, but 19.1% of mothers thought that it has not necessary to take such protective measures. The responses given by the fathers to this question were very similar to the mothers.

**Table 7:** Parents were asked if they would take any preventive measures for themselves and their children if they had to go to the dentist during the pandemic.

**Sociodemographic factors**	**Yes (%)**	**No (%)**	**Chi-square**	***P*-value**
**Parenthood relationship**				
Mother	161 (80.9)	38 (19.1)	0.001	0.973
Father	46 (80.7)	11 (19.3)		
**Age groups (years)**				
<30	46 (88.5)	6 (11.5)	4.855	0.088
30–40	113 (81.9)	25 (18.1)		
>40	48 (72.7)	18 (27.3)		
**Level of education**				
Primary—High School	59 (73.8)	21 (26.3)	3.800	0.051
Graduate—Postgraduate	148 (84.1)	28 (15.9)		
**Place of residence**				
City	69 (82.1)	15 (17.9)	0.133	0.715
Countryside	138 (80.2)	34 (19.8)		
**Occupation**				
Health professionals	29 (85.3)	5 (14.7)	0.498	0.480
Non-health professionals	178 (80.2)	44 (19.8)		
**Income (US dollars)**				
<511	26 (74.3)	9 (25.7)	1.630	0.443
511–1.538	135 (80.8)	32 (19.2)		
>1.538	46 (85.2)	8 (14.8)		

**Figure 5:**
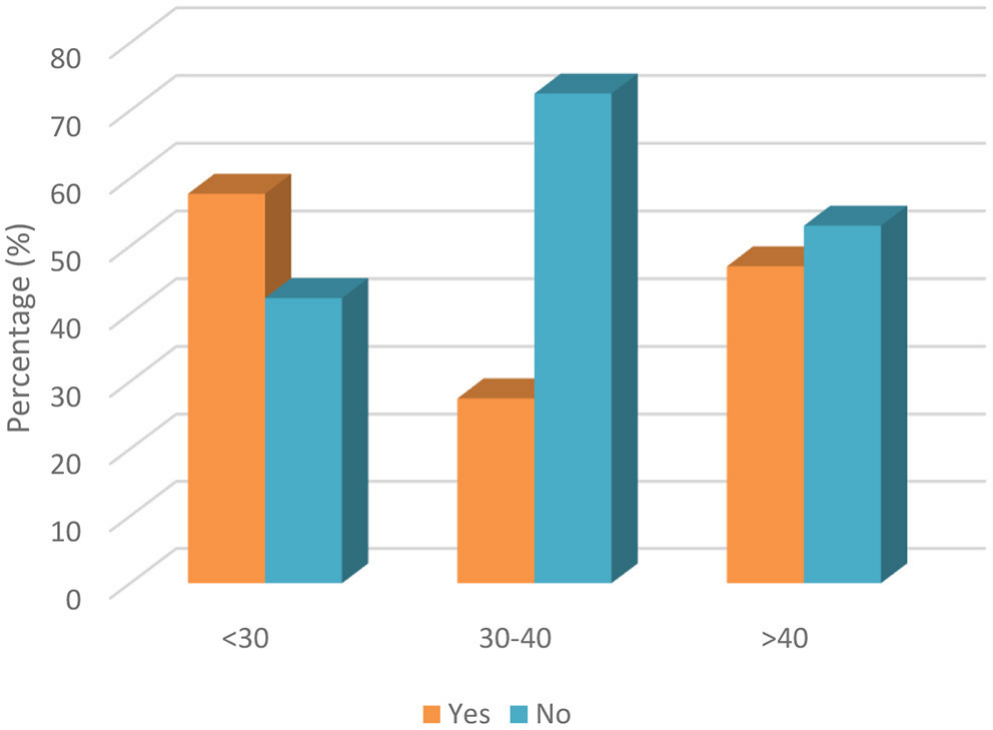
Response of parents to whether they took any additional precautions for their children' s oral healthcare during COVID-19 based on age groups.

The statistical analysis of whether parents took additional dental precautions for their children during that period is shown in Table 8. The only significant difference was found between the three age groups. The percentage of positive responses for any extra precautions in the <30, 30–40, and >40 age groups were 32.7, 13.0, and 16.7%, respectively (Figure 5). No significant difference was found in terms of parenthood relationships, while 80.9% of the mothers reported that they did not take any extra precautions regarding their child's oral health during this period, and only 19.1% were sensitive to this issue. Additionally, when the responses given by the fathers were examined, it was observed that they were very similar to the mothers with close percentages.

**Table 8:** Response of parents regarding whether they took any additional precautions for their children during the COVID-19 pandemic.

**Sociodemographic factors**	**Yes (%)**	**No (%)**	**Chi-square**	***P*-value**
**Parenthood relationship**				
Mother	38 (19.1)	161 (80.9)	0.770	0.380
Father	8 (14.0)	49 (86.0)		
**Age groups (years)**				
<30	17 (32.7)	35 (67.3)	9.995	**0.007**
30–40	18 (13.0)	120 (87.0)		
>40	11 (16.7)	55 (83.3)		
**Level of education**				
Primary—High School	14 (17.5)	66 (82.5)	0.017	0.895
Graduate—Postgraduate	32 (18.2)	144 (81.8)		
**Place of residence**				
City	15 (17.9)	69 (82.1)	0.001	0.974
Countryside	31 (18.0)	141 (82.0)		
**Occupation**				
Health professionals	8 (23.5)	26 (76.5)	0.822	0.364
Non-health professionals	38 (17.1)	184 (82.9)		
**Income (US dollars)**				
<511	32 (91.4)	3 (8.6)	2.757	0.252
511–1.538	133 (79.6)	34 (20.4)		
>1.538	43 (79.6)	11 (20.4)		

*Bold shows statistical significance (p < 0.05)*.

The parents were asked if they thought their children could become infected when receiving dental treatment. An overwhelming majority of the parents (73.4% of mothers and 75.4% of fathers) were worried about them being infected during dental treatment and avoided these procedures. The rest of the parents (26.6% of mothers and 24.6% of fathers) did not have the same opinion.

## Discussion

Even though numerous studies related to dentistry have been conducted during the COVID-19 pandemic, studies on parents and the pediatric dentistry field are very rare. When the sociodemographic features of the participants were analyzed, it was found that the vast majority of parents were generally mothers (77.7%). The clearly shows that mothers are more likely to be interested in their children's oral health status during the pandemic period and this was consistent with previous studies (21–24).

The knowledge of parents (67.8% of mothers and 68.4% of fathers) about whether children are more resistant to COVID-19 was found to be good, which is the major finding in the present research. This finding of the study was consistent with that reported by Abuhammad (18). On the other hand, the knowledge of parents (89.4% of mothers and 77.2% of fathers) about transmission of COVID-19 by air droplets during dental treatment was also found to be extremely high in the present research. However, the knowledge of mothers about this issue was detected to be higher compared to fathers. In addition to this comparison, the parental attitudes of mothers (81.9%) about taking their children for dental treatment during COVID-19 was detected to be higher compared to fathers (59.6%). These findings of the study could be justified by the fact that mothers are more concerned and feel anxious about COVID-19 and the health of their children. The results of the study by Farsi et al. (25) study were partially consistent with this result of the current study. Additionally, it is possible to state that a partially negative correlation between the knowledge of parents about COVID-19 transmission and attitudes toward taking dental treatment has been found, different from Li et al.'s study (17).

Today, it is widely known that aerosol-generating procedures during dental treatments that cause infective pathogens could lead to accumulation on surrounding dental surfaces (26). Dental patients who have been diagnosed with or are suspected of having COVID-19 are recommended to delay their appointment, apart from urgent conditions (27). For this reason, it is important that parents are aware about which conditions require urgent dental treatment. According to the results of the current study, the awareness of health professional parents (70.6%) about urgent dental procedures was found to be better compared to non-health professional parents (49.5%). Generally, the participants who were health professionals considered traumatic dental injuries and swelling as urgent conditions, while non-health professionals believed that crowded teeth and dental scaling were urgent dental conditions. Besides these situations, the vast proportion of parents did not take any additional precautions for their children's oral healthcare during the COVID-19 pandemic. Only a large proportion of health professional parents (61.8%) exhibited an increased level of concern for oral health during the pandemic.

Another important issue that should be discussed and compared is “the importance and effectiveness of web-based surveys during the COVID-19 pandemic.” Web-based surveys have various positive aspects such as minimum human resources, less stationery costs, time-saving benefits, ease of use for participants and researchers, rapid analysis, and less effort compared with data being downloaded directly to a database. Especially in this period, conducting traditional surveys face-to-face would increase the risk of transmission of the virus. Due to these reasons, the web-based survey method was preferred and designed for this study.

Transmission of the COVID-19 has caused significant difficulties and reduced people's quality of life in countries all over the world. The speed of manifestation of symptoms of the disease and its outcomes vary across healthcare systems and economies (28).

Prevention and public awareness of COVID-19 are important aspects of disease control. Therefore, the lack of knowledge of infectious diseases may cause low detection rates of the virus (29). According to the findings of this study, it is clear that the level of knowledge of parents about transmission routes of the COVID-19 virus was very high, similar to the results of Surme et al. (30) and Ge et al. (26). Also, Surme et al. (30) compared the education level and knowledge of transmission routes of COVID-19 similar to the present study and they reported a significant difference between them. On the other hand, with regard to transmission by air droplets, a statistical difference was only found in parenthood relationship and occupation; no significant differences were found between the education level of parents according to their level of knowledge about transmission routes (30). In the present study, when the occupational groups and droplet transmission were compared, it was found that healthcare workers (100%) had more information than other sectors (84%). The reason for this may be that health workers are at the forefront of the fight against the disease during the COVID-19 pandemic. In terms of parenthood relationship and transmission by air droplets, the results showed that mothers had more knowledge about such risks since they are more concerned about the healthcare of children in Northern Cyprus.

Consistent with Dong et al.'s study (31), which reported that children in different age groups are susceptible to COVID-19 regardless of gender, and the clinical manifestations of COVID-19 positive children are less severe than those of adult, in the present study, this assertion was supported by the fact that the majority of parents thought that children were resistant to COVID-19.

Sun et al. (16) reported that 91.89% of parents thought that their children can be easily infected during dental treatment. On the other hand, in another study, only 25.2% of parents stated that their children could be infected during dental treatment procedures (30). In this study, 74.4% of the families agreed with this statement, which was similar to the finding of Sun et al. (16) on this issue. The reason for this difference is thought to be due to the fact that both our study and Sun et al.'s (16) study were carried out recently in 2020. On the other hand, Surme et al.'s (30) study was conducted at a later time in 2021, and they recommended that dental treatments be postponed except for emergency treatments by taking contagion precautions.

With respect to the responses of parents to the question regarding the conditions that require emergency dental treatment, no significant differences were found between parenthood relationships, age groups, and level of education. A significant difference was only found between occupation and income. Healthcare professionals seem less knowledgeable about emergency dental treatments, which may be due to the fact that healthcare professionals know that even the smallest dental problem can cause serious consequences, and they are not worried about taking their children to the dentist even during the COVID-19 pandemic. Furthermore, as the income level of the parents' decreases, the level of knowledge about emergency dental treatments increases. The reason for this is thought to be that families with low income cannot afford every dental treatment, so they do more research on the dental health of children.

Concerning this outcome, a similar question was asked by Surme et al. (30) and they only found a statistically significant difference at the education level, reporting that university graduates would have all dental procedures performed for their children during the COVID-19 pandemic. This means they were not aware of the procedures that required emergency treatment. In contrast to the earlier findings of Sun et al. (16), in the present study, it was suggested that there were no significant differences between female and male parents with respect to the conditions that require emergency treatment. Also, parents with a primary-high school level of education were less knowledgeable and parents with a graduate-postgraduate degree were relatively more knowledgeable in relation to our results, and it is seen that according to income, low-income individuals have more information about conditions that require emergency treatment. The reason for this may be due to the high socio-cultural level in our country.

## Conclusion

Although most parents had adequate knowledge about COVID-19 and the aerosol transmission pathway during dental visits, the awareness of parents about the conditions that require urgent dental treatment was very low. Also, parents did not take additional adequate precautions their children's oral healthcare during the pandemic period. As a result, it may be concluded that parents have been affected by the COVID-19 pandemic and this has impacted their attitudes toward dental visits and oral health management.

Further steps should be taken to strengthen parents' motivations for home oral healthcare. More studies are needed to assess the continuing impact of the COVID-19 pandemic on parents' attitudes toward and knowledge about dental procedures. In the light of current data “Tele-dentistry” should be proposed as an approach that can be adopted in the future to increase parents' oral healthcare management of their children as well as to determine which conditions require urgent dental treatment during the pandemic in a rapid and reliable manner.

Significant Statement: This study identified the important role that parents play in their children's oral health during the pandemic period, which can be beneficial for decreasing dental visits to avoid cross infections. This study will help researchers to uncover the critical areas of parental attitudes and COVID-19 relationship that many researchers were not able to explore. Tele-dentistry can be used as a supportive tool for dentists in diagnosing urgent dental cases. Thus, parents will understand whether their children need treatment without going to dental clinics, and this will prevent transmission by preventing unnecessary crowding in clinics. Hence, a new theory on tele-dentistry may be arrived to improve the oral health status of children by parents during the pandemic.

## Data Availability Statement

The raw data supporting the conclusions of this article will be made available by the authors, without undue reservation.

## Author Contributions

Material preparation and data collection were performed by AE. Statistical analysis was performed by OT. The first draft of the manuscript was written by AE and AI and commented on previous versions of the manuscript. All authors contributed to the study's conception, design, and read and approved the final manuscript.

## Conflict of Interest

The authors declare that the research was conducted in the absence of any commercial or financial relationships that could be construed as a potential conflict of interest.

## Publisher's Note

All claims expressed in this article are solely those of the authors and do not necessarily represent those of their affiliated organizations, or those of the publisher, the editors and the reviewers. Any product that may be evaluated in this article, or claim that may be made by its manufacturer, is not guaranteed or endorsed by the publisher.
